# The Influence of Mineral Additives on Aggregate Reactivity

**DOI:** 10.3390/ma18010007

**Published:** 2024-12-24

**Authors:** Grzegorz Rogojsz, Tomasz Rudnicki

**Affiliations:** Faculty of Civil Engineering and Geodesy, Military University of Technology, 2 Gen. Sylwestra Kaliskiego Str., 00-908 Warsaw, Poland; grzegorz.rogojsz@wat.edu.pl

**Keywords:** mineral additives, alkaline reactivity, standard mortar

## Abstract

In this article, the authors present the results of their research on assessing the effect of selected mineral additives on the alkaline reactivity of aggregates. The main objective of this research was to check whether the reactivity of aggregates that do not meet the standard requirements can be reduced. Due to the decreasing availability of crushed aggregates and the decreasing resources of sand used for cement concrete road surfaces, solutions should be sought that allow the use of lower-grade aggregates. Among the available mineral additives, dense microsilica, white microsilica, limestone flour, glass flour, basalt flour, and glass granulate were selected. Laboratory tests were carried out in accordance with the requirements for testing the alkaline reactivity of road aggregates in NaOH solution applicable in Poland. The tests included the use of mineral additives in the amounts of 10% and 20%. Based on the research conducted, it was observed that the most beneficial effect was obtained with the addition of white microsilica, for which a decrease in aggregate reactivity was observed by 76.7% for 10% of the additive and 95.8% for 20% of the content. The least beneficial effect, on the other hand, was the use of compacted microsilica, for which an increase in alkaline reactivity was observed by 9.3% for 10% of the additive and 20.9% for 20% of the additive. The research conducted shows that the alkaline reactivity of the aggregate can be reduced, due to which it is possible to use reactive aggregates for the construction of road surfaces made of cement concrete.

## 1. Introduction

Mineral additives, which are common by-products of various industries, can have a significant impact on the alkaline reactivity of concrete, especially in the context of the so-called alkaline–silica reaction (ASR). This reaction is a chemical process between alkaline ions (e.g., sodium and potassium ions) from cement and reactive silica species present in some aggregates [1,2,3,4,5,6]. The reaction results in the formation of products that swell in the presence of moisture, leading to serious damage to the concrete, such as micro-cracks, structural weakening, and loss of strength [7,8,9,10,11,12]. Fly ash and blast furnace slag have a very significant impact, reducing the risk of alkaline–silica reactions (ASRs) due to the reaction of alkaline ions from cement and reactive silica in the aggregate, causing swelling and damage to the concrete [13,14,15]. Mineral additives containing aluminum and silicon compounds (e.g., fly ash, blast furnace slag) can react with alkaline ions, binding them into insoluble phases. This leads to a decrease in the pH of the pore solution, which slows down the ASR. Mineral additives such as fly ash, metakaolin, colloidal silica, or granulated blast furnace slag can significantly reduce the alkaline reactivity of concrete [16]. An interesting phenomenon described in the literature is the dilution effect, i.e., mineral additives reduce the total clinker content in cement, which reduces the amount of alkali. As a result, concrete is less susceptible to alkali–silica reactions because the concentration of alkali responsible for initiating this reaction is reduced [17,18,19,20]. In addition, we dealt with a modification of the chemical composition of cement phases. Mineral additives can also modify the chemical composition of cement phases, limiting the formation of minerals with high alkaline reactivity. Another mechanism of action of mineral additives is to lower the pH of the pore solution. This prompted us to look for other available mineral additives to reduce the reactivity of mortars containing potentially reactive aggregates.

Analyzing the literature, we can find studies on the effect of fly ash in specific concrete applications [21] (in terms of strength and durability) and economic aspects (in terms of reuse of waste materials from industrial processes). A significant part of mineral additives also enters the pozzolanic reaction. In the case of fly ash and other pozzolanic additives, the pozzolanic reaction occurs, consisting of the binding of calcium hydroxide (Ca(OH)_2_), one of the products of cement hydration, to insoluble silicon and calcium compounds [22,23,24,25]. This reaction reduces the availability of Ca(OH)_2_, which indirectly reduces alkaline reactivity because the calcium ion stabilizes the alkaline–silica reaction.

Mineral additives also have a significant effect on the properties of the mortar, improving its durability, strength, and resistance to external factors. The main aspects of the action of these additives include, above all, increasing compressive strength. Additives such as colloidal silica, fly ash, or blast furnace slag enter into pozzolanic reactions, creating additional hydration products that improve the structure of the mortar. The influence of mineral additives also improves durability by sealing the mortar and increasing its resistance to moisture and aggressive ions (e.g., chlorides and sulfates). As a result, the mortar becomes more resistant to environmental factors and chemical degradation, which extends its service life. This proves that selected additives can have an impact on increasing compressive strength [26,27,28,29,30,31,32]. Another area of action of additives is the reduction in mortar and concrete shrinkage by reducing the amount of heat generated during cement hydration, which reduces the risk of plastic shrinkage and cracking. This also increases the adhesion and cohesion of the cement matrix [33,34,35,36,37].

Some additives, such as colloidal silica, improve the rheological properties of the mortar, which makes it easier to lay and increases adhesion to the substrate. Mortar with such additives is less susceptible to the segregation of components and is more homogeneous. Increased resistance to water and penetration of aggressive ions: mineral additives reduce the porosity of concrete, which limits the permeability of water and chemical compounds (e.g., chlorides and sulfates), which can accelerate the corrosion of reinforcement and damage to the concrete structure. The less permeable structure of concrete means that it is more resistant to corrosion and frost cracking. The positive effect of additives can also be observed through an increased resistance to high temperatures. Additives such as metakaolin can improve the resistance of mortar to high temperatures. Metakaolin is less susceptible to thermal degradation than standard clinker, which makes the mortar more resistant to fire and variable thermal conditions [38]. The last element of the impact of additives on mortar and cement concrete is undoubtedly a significant improvement in aesthetics: mineral additives affect the appearance of the mortar and can give it a more uniform and lighter color, which is desirable in prefabricated elements and in finishing works.

In summary, the use of appropriate mineral additives is one of the key methods of reducing the alkaline reactivity of concrete and improving its durability. Properly selected additives lower the pH of the pore solution, bind alkaline ions, reduce the number of capillary pores, and reduce the clinker content, which significantly reduces the risk of alkali–silica reactions. Additionally, they have a beneficial effect on the mortar in terms of strength, durability, aesthetics, and resistance to various external factors. The appropriate selection of mineral additives can, therefore, significantly improve the properties of the mortar, increasing its quality and suitability for various construction applications.

## 2. Materials and Methods

### 2.1. Materials

**Figure 1:**
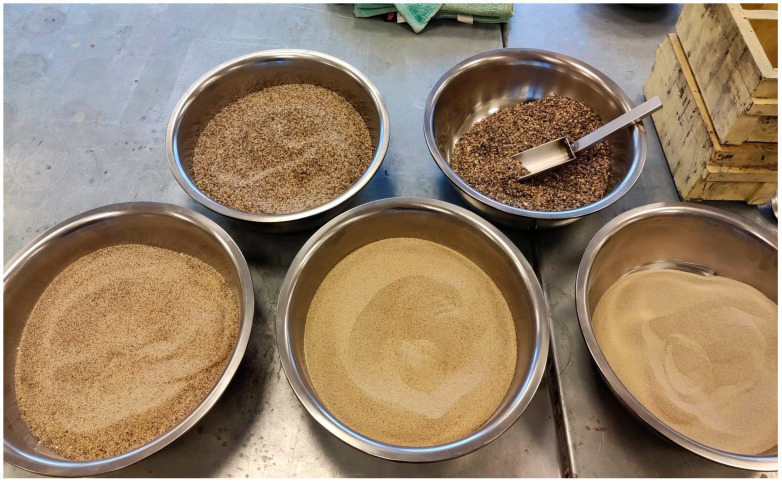
Fractionated sand at 0/2 mm.

In order to assess the effect of mineral additives on the alkaline reactivity of the aggregate, river sand with a grain size of 0/2 mm was used. Since the natural grain size of the sand did not meet the requirements specified in [39], it was fractionated into individual fractions (Figure 1) and then mixed in accordance with the grain size requirements presented in Table 1.

The cement class CEM I 42.5 R, hereinafter referred to as CEM I, was used to prepare the samples, with the parameters presented in Table 2.

**Figure 2:**
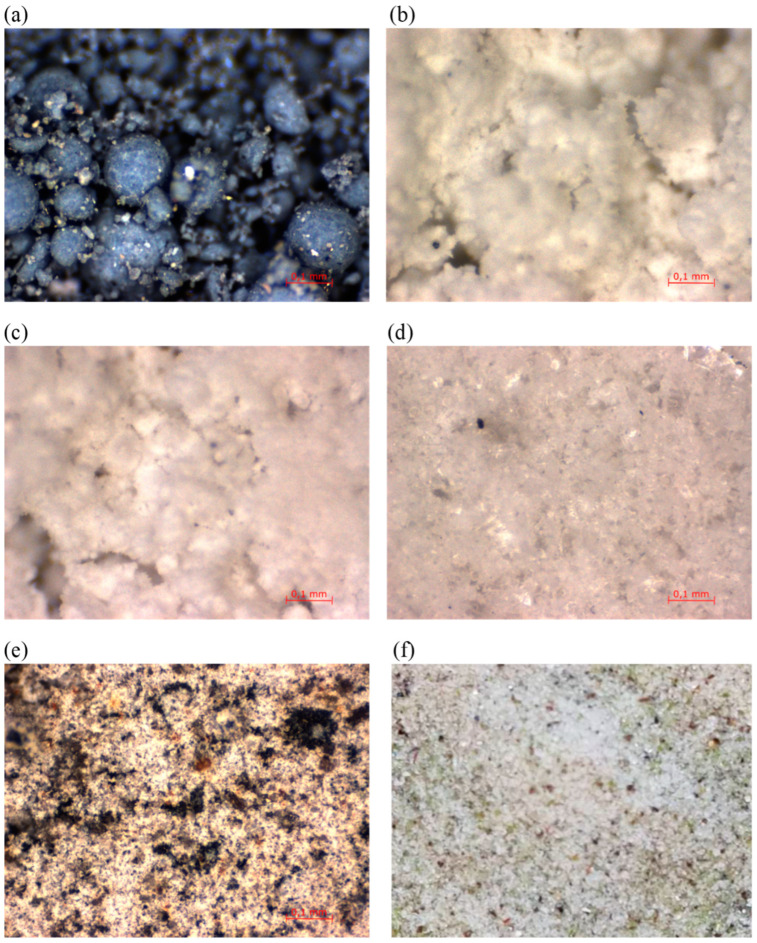
Microscopic photos of mineral additives: (**a**) compacted microsilica, (**b**) white microsilica, (**c**) limestone flour, (**d**) glass flour, (**e**) basalt dust, and (**f**) glass granulate.

**Figure 3:**
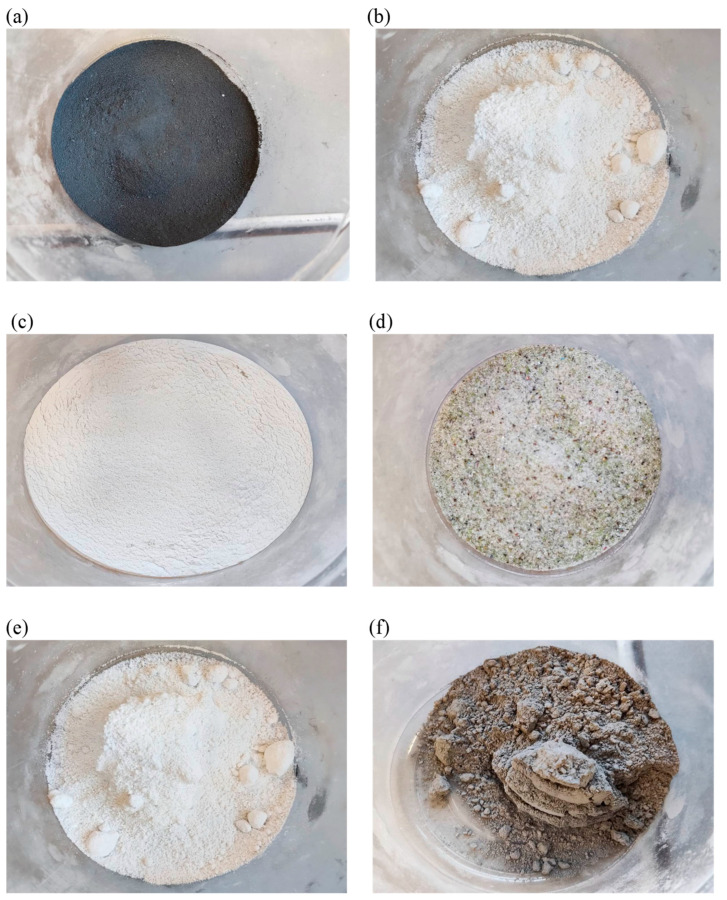
Mineral additives: (**a**) compacted microsilica, (**b**) white microsilica, (**c**) glass powder, (**d**) glass granulate, (**e**) limestone powder, and (**f**) basalt dust.

The mineral additives used were compacted microsilica (MkZ) from Mikrosilika Trade in the form of silica dust generated in arc furnaces during the production of metallic silicon and ferrosilicon alloys, and white microsilica (MkB), which is a by-product of zirconium silicate production. Another additive was limestone flour (MW), which is used as a filler for mineral–asphalt mixtures created by drying and grinding limestone, the main component of which is calcium carbonate. The influence of glass waste generated on the basis of construction glass with a hardness of 6–7 on the Mosh scale in the form of glass flour (MS) and glass granulate (GS) was also examined. Another additive analyzed was basalt dust (PB), also called basalt flour, which is waste generated during the processing of aggregate for the production of mineral–asphalt mines. The chemical composition of the individual additives is presented in Table 3, and the additives are shown in Figure 2 and Figure 3.

Compacted microsilica and white microsilica are characterized by a homogeneous structure in the form of spherical grains with a maximum size below 0.003 mm. Compacted microsilica in loose form creates clusters of grains of 0.1–0.2 mm in size, as visible in Figure 1, created by the agglomeration of small particles, which immediately disintegrate after contact with water. Limestone flour consists of non-homogeneous grains, mainly below 0.06 mm in size, which do not interconnect with each other, creating a loose material in the form of dust, as shown in Figure 2c. In contrast to limestone flour, glass flour is characterized by a very homogeneous structure, with grains below 0.06 mm in size (Figure 2d). Basalt dust, like limestone flour, is formed by non-homogeneous grains of crushed aggregate with a size below 0.08 mm (Figure 2e). Glass granulate is composed of irregular grains up to 0.5 mm in size, as shown in Figure 2f.

### 2.2. Methods

Samples for laboratory tests were prepared in accordance with the procedure described in the instructions [39] in the form of bars with dimensions of 25 mm × 25 mm × 285 mm, with 3 pieces for each additive. In the first stage of the tests, 10% of the cement content was replaced with a mineral additive, and in the second stage, 20% of the cement content was replaced with a mineral additive. In order to compare the effect of individual additives on the alkaline reactivity of the sand used in the tests, reference samples without mineral additives were also prepared. The composition of the individual mixes for stages 1 and 2 is presented in Table 4, and the description of the symbols of the test samples used is presented in Table 5.

After preparation, the test samples were conditioned in a humidity chamber at a temperature of 20 ± 1 °C and a humidity of no less than 90% for a period of 24 ± 2 h. After this time, the samples were unmolded, and the length of the samples was measured, constituting a dimensional base with an accuracy of 0.001 mm. After the initial measurement, the test samples were placed in a container filled with distilled water and placed in a thermostatic chamber at a temperature of 80 ± 2 °C for a period of 24 h. After this time, a zero measurement was performed, and the test samples were placed in a container containing a 1 molar NaOH solution at a temperature of 80 ± 2 °C. The length of the samples was measured after 1, 7, 10, 14, and 16 days of storage in the NaOH solution at a temperature of 80 ± 2 °C. The measurement procedure used was based on the instruction PB-1-18 [40] developed by the General Directorate for National Roads and Motorways in Poland. The evaluation of the effect of mineral additives on the reactivity of the aggregate was carried out based on the results of the average change in the length of the mortar samples after 14 days of storage in 1 molar NaOH solution, as shown in Table 6.

## 3. Results

### 3.1. Determination of the Reactivity of the Mortar for Stage 1 (10% Addition)

The difference between the zero measurements of the sample length and the measurement of the length at each storage period was calculated with an accuracy of 0.001% for the effective distance between the ends of the plugs placed in the mortar. The change in the length of the sample tested was calculated using the following equation:

Change in sample length [%]     = 100 × (L_n_ − L_0_)/G (1)

where

L_n_—sample length after “n days” [mm], where n is the number of days from the zero measurement;
L_0_—zero sample length [mm];
G—distance between the inner ends of the plugs in the mortar samples [mm] with an accuracy of 0.1 mm

**Figure 4:**
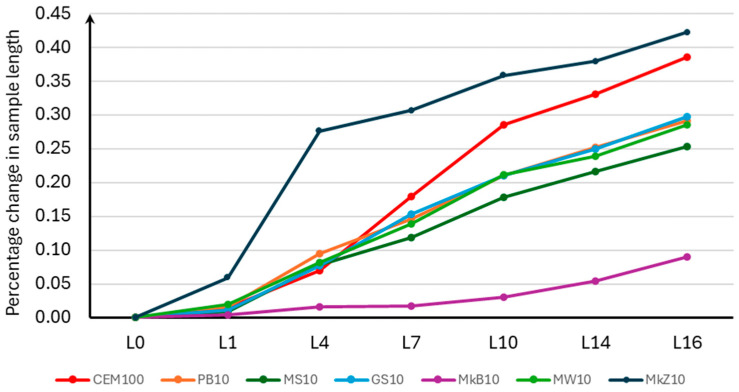
Average percentage change in length of samples for Stage 1.

The results of the percentage change in the length of the samples for Stage 1 are presented in Table 7, and the average values are presented in Figure 4.

Since the observed measurement error was less than 0.005% of the increase in THE sample length, it is not plotted on the graph below.

Analyzing the obtained results, it should be stated that mineral additives have a significant influence on the results of potential alkaline reactivity. The study analyzed the effectiveness of the influence of mineral additives on the change in the length of the tested samples. All analyzed additives, except for compacted microsilica, had a positive effect on the change in the length of the samples compared to the control mixture without additives. The most beneficial was the use of white microsilica, which reduced the percentage increase in the length of the samples by 76.6%. Therefore, the use of white microsilica (MkB10) reduced the alkaline reactivity category of the aggregate used from R2 to R0. The least beneficial effect was the use of compacted microsilica (MkZ10) because it increased the percentage increase in the length of the samples by 9.5% without affecting the change in the reactivity category of the aggregate. The remaining mineral additives in the form of limestone flour (MW10), glass flour (MS10), glass grit (GS10), and basalt dust (PB10) had a similar effect on the reduction in the percentage increase in the length of the samples, limiting their increase by 25.7%, 34.2%, 22.9%, and 24.3%, respectively, which allowed the reactivity class to be changed from R2 to R1.

### 3.2. Determination of Mortar Reactivity for Stage 2 (20% Addition)

**Figure 5:**
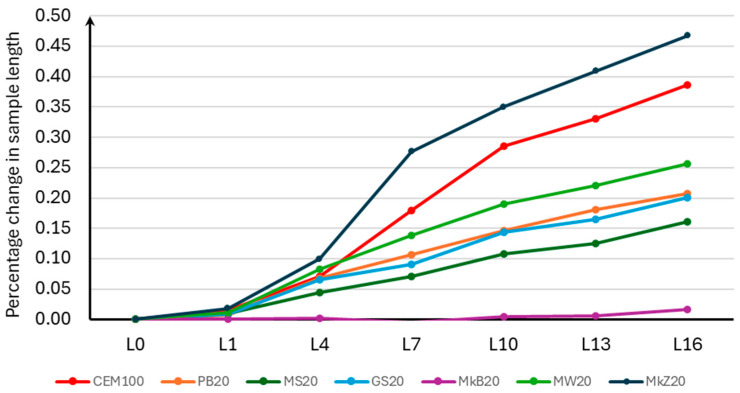
Average percentage change in length of samples for Stage 2.

As in the case of Stage 1, for samples with 20% mineral additives, the difference between the zero measurements of the sample length and the measurements of the length at each storage period was calculated to the nearest 0.001% of the effective distance between the ends of the plugs placed in the mortar, according to Equation (1). The results of the percentage change in the length of the samples for Stage 2 are presented in Table 8, and the average values are shown in Figure 5.

Since the observed measurement error was less than 0.005% of the increase in the sample length, it is not plotted on the graph below.

Analyzing the presented results, it should be noted that the 20% content of mineral additives also had a significant influence on aggregate alkaline reactivity. Similarly to the previous case, the most beneficial influence on limiting the percentage increase in the sample length was that of white microsilica (MkB20). Increasing its share in the mixture allowed us to practically eliminate the phenomenon of a sample length increase. Limiting the percentage increase in the sample length by 95.7% also allowed us, in this case, to change the alkaline reactivity class from R2 to R0. The use of compacted microsilica caused a percentage increase in the sample length of 21.1% to the value of 0.4094%. This change did not adversely affect the decrease in the alkaline reactivity category of the aggregate. However, it should be noted that in the case of testing the length increase after 16 days, it was already 0.4679%, which allowed us to classify the aggregate as very strongly reactive with category R3. For the 20% additive content, in the case of the length increase test after 14 days, the use of glass flour (MS20) also allowed us to reduce the reactivity category from R2 to R0, and the limitation of the percentage increase in the sample length was 67.7%; however, considering the results after 16 days, the limitation of the percentage increase in the sample length was 58.1%, which allowed us to change the aggregate reactivity class from R2 to R1. Basalt dust (PB20) and glass granulate (GS20) have almost the same effect on the limitation of the percentage length of the sample by 46.2% and 47.9%, respectively. Also, in this case, we dealt with a change in the alkaline reactivity category of the aggregate from R2 to R1. The mineral additive in the form of limestone flour limits the percentage increase in the length of the samples to the lowest extent, only by 33.6%, which allowed us to change the alkaline reactivity category of the aggregate used from R2 to R1.

**Figure 6:**
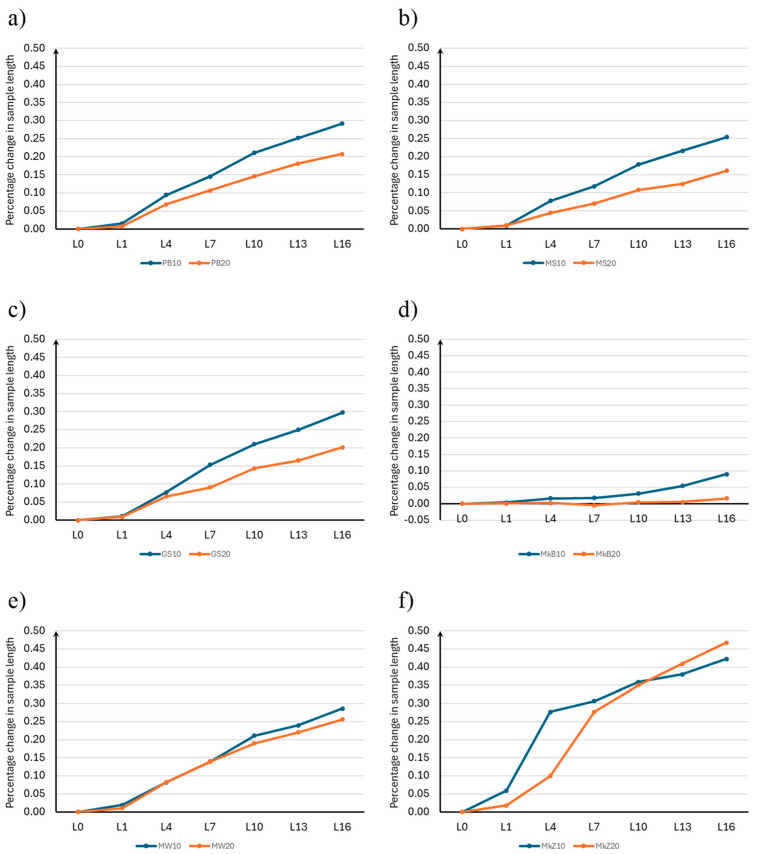
Summary of the average percentage change in sample length for Stages 1 and 2: (**a**) basalt dust, (**b**) glass powder, (**c**) glass granulate, (**d**) white microsilica, (**e**) limestone powder, and (**f**) compacted microsilica.

Figure 6 presents the results of the average percentage change in the length of the samples for each mineral additive, comparing the 10% and 20% contents of additives.

Comparing the use of 10% and 20% mineral additives, it should be noted that in the case of basalt dust, glass flour, glass grit, and white microsilica, increasing the content of the additive had a significant beneficial effect on limiting the percentage increase in the sample length, which increased by about 0.1%. In the case of limestone flour, doubling its share in the mixture did not cause significant differences in the final results of the percentage increase in the length of the samples, which was only 0.03%. Increasing the content of the additive in the form of compacted microsilica in the initial phase of the study had a more beneficial effect on the increase in the length of the samples; however, after the 10th day of conditioning, greater increases in the length of the samples occurred than in the case of 10% compacted microsilica additive. The difference in the obtained results was similar to that for limestone flour and was less than 0.05%.

**Figure 7:**
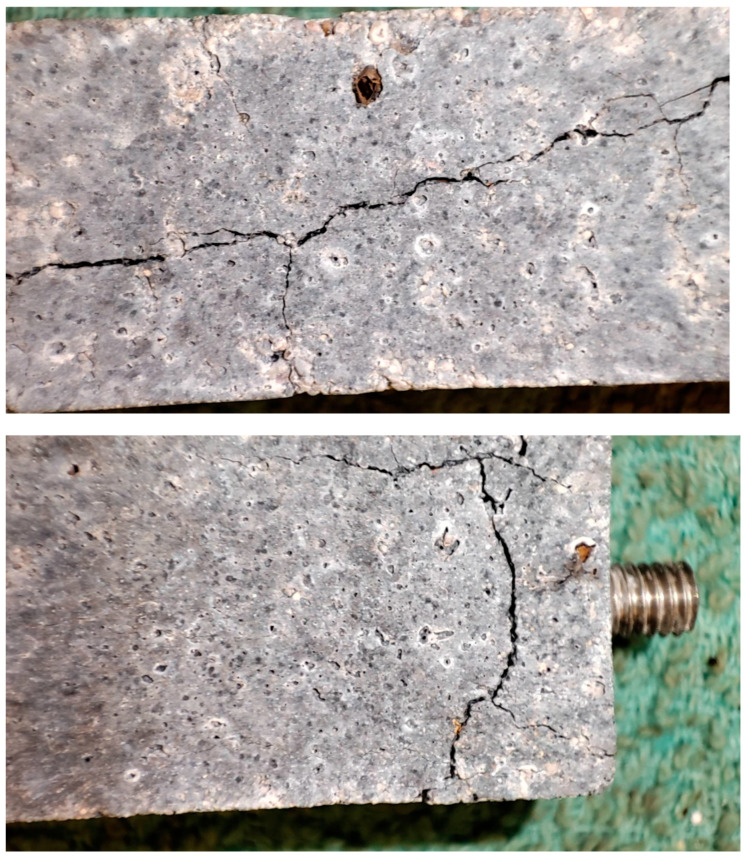
Cracks in samples with 20% addition of compacted microsilica.

The unfavorable effect of compacted microsilica on the tested samples was also observed in the form of cracks and scratches appearing on the samples, as shown in Figure 7.

## 4. Discussion

As mentioned at the beginning of this article, the course of the alkaline reactivity phenomenon is influenced by many factors, and three basic alkaline reactions can be distinguished:

- The reaction between amorphous silica (a component of aggregates) and alkalis:

SiO_2_ + 2NaOH → Na_2_SiO_3_ + H_2_O

- The reaction between alkalis and carbonates (mainly in the case of aggregates from carbonate rocks—limestones and dolomites):

CaMg(CO_3_)_2_ + _2_NaOH^−^ → Mg(OH)_2_ + CaCO_3_ + Na_2_CO_3_

Na_2_CO_3_ + Ca(OH)_2_ → 2NaOH + CaCO_3_

- The reaction between alkali and poorly crystallized forms of silicates.

As a result of the alkali–aggregate reaction, gel shells are formed around the aggregate grain, which swells under the influence of water, causing stress in the concrete. These stresses can cause scratches or cracks.

Analyzing the reactions presented above, it can be concluded that mixtures with an increased content of silica or calcium carbonate should be more susceptible to alkaline reactivity. Among the analyzed mineral additives, compacted microsilica and white microsilica are characterized by a SiO_2_ content above 80% and 95%, respectively.

In the case of the compacted microsilica additive at the amount of both 10% and 20%, an increase in alkaline reactivity was obtained, which was observed at 9.3% and 20.9%, respectively. The authors of the work [41] using microsilica at the amount of 10% and 20% achieved a reduction in sample expansion by about 50%; however, this is mainly due to the use of slag as an additive at the amount of 60% and 70%. For the additive in the form of white microsilica, which has the highest SiO_2_ content, a decrease in alkaline reactivity was observed, amounting to 76.7% for the content of 10% white microsilica and 95.8% for the content of 20%.

Also, in the case of the mineral additive in the form of limestone flour, which is characterized by the highest CaCO_3_ content of over 93%, a decrease in alkaline reactivity was observed for both 10% and 20% of the additive, which was obtained at 25.8% and 33.7%, respectively, and was also confirmed in the experimental studies carried out by the authors of these works [42,43].

The total content of alkalis in concrete also affects alkaline reactivity. It should, therefore, be assumed that mortars with a higher Na_2_Oeq content should be characterized by a higher alkaline reactivity. In the experimental studies conducted, the highest Na_2_O content, exceeding 14%, was characterized by glass flour and glass granulates. However, for both additives, a decrease in the alkaline reactivity of the aggregate was obtained. For the content of additives at the amount of 10%, it was 34.4% for glass flour and 26.1% for glass grit. On the other hand, for the content of additives at the amount of 20%, the decrease in alkaline reactivity was 58.2% for glass flour and 47.9% for glass grit. It can, therefore, be stated that the increase in the alkali content in the mixture contributed to the decrease in the alkaline reactivity of the aggregate. According to the authors of the work [16], the additives that affect the decrease in the alkaline reactivity of aggregates are mainly zeolites, fly ash, or lithium compounds.

## 5. Conclusions

Based on the tests conducted involving the introduction of mineral additives at the amount of 10% and 20% to the standard mortar as a cement substitute, the following conclusions can be drawn:

(1) Compacted microsilica, which is a material commonly used in UHPC concretes, increases the alkaline reactivity of aggregates. In the discussed tests, the reactivity was 9.3% for the 10% additive content and 20.9% for the 20% additive content, respectively. It should, therefore, be stated that compacted microsilica is not suitable for reducing the alkaline reactivity of aggregates intended for road surfaces.
(2) The addition of white microsilica at the amounts of 10% and 20% caused a decrease in the alkaline reactivity of the aggregate by 76.7% and 95.8%, respectively, which contributed to reducing the aggregate reactivity class from R2 to R0. This is very beneficial because reactive aggregates can be used to build road surfaces made of cement concrete. This directly contributes to the possibility of using aggregates for road construction, which until now has not met the standard requirements. Nevertheless, there is a risk of increased shrinkage of the concrete mix, which will also constitute a further stage of the authors’ research.
(3) A very positive effect can be observed after adding limestone flour, which is characterized by the highest CaCO_3_ content of over 93%, and an additive in the form of glass flour and glass granulate, characterized by an increased Na_2_Oeq content. The research conducted shows that the use of the above additives also has a beneficial effect on reducing alkaline reactivity and aggregates from carbonate rocks and will not necessarily enter into a reaction between alkalis and carbonates. It is, therefore, possible to use limestone aggregates mainly in the form of limestone flour as a substitute for cement in order to reduce the alkaline reactivity of the aggregate.
(4) The results obtained in the research constitute an important direction in the scope of the possibilities of using mineral additives to minimize the alkaline reactivity of aggregates, and additionally, as was confirmed in the results presented in the work [32], by using mineral additives, it is possible to reduce the cement content in the mortar, which also has a positive effect due to environmental aspects and reducing carbon footprint.
(5) In Poland, over 70% of sand mines are characterized by alkaline reactivity at the level of R1 or R2; therefore, due to the use of the above-mentioned additives, this level can be significantly reduced, and the amount of sand used in the construction of roads and bridges can be increased.

## Figures and Tables

**Table 1:** Required sand grain size for alkali reactivity testing.

Fraction [mm]	Contents [%]
2–4	10
1–2	25
0.5–1	25
0.25–0.5	25
0.125–0.25	15

**Table 2:** Properties of cement CEM I 42.5 R.

Parameter	Unit	Value
Specific surface area	cm^2^/g	4124
Start of setting time	Min	184
End of setting time	Min	242
Change in volume	Mm	1.0
Compressive strength		
After 2 days	MPa	30.1
After 28 days	MPa	60.2
Content of SO_3_	%	2.95
Content of Cl	%	0.089
Insoluble residue	%	0.57
Loss of ignition	%	3.33

**Table 3:** Chemical composition of the additives used.

Component	Unit	MkZ	MkB	MW	MS	PB	GS
SiO_2_	%	>80.0	>94.0	3.5	>65.0	38.2	>65.0
CaO	%	<3.5	<1.0		>8.0	15.2	>8.0
ZrO_2_	%		<4.0				
CaCO_3_	%			93.0			
FeO_3_	%		<1.0	0.3	<0.2	15.9	<0.2
MgO	%			0.7	<0.4	7.7	<0.4
SO_3_	%	<4.0				0.2	
Na_2_O	%	<8.0			>14.0	2.9	>14.0
Al_2_O_3_	%		<1.0		2.0	12.7	2.0
Cl^−^	%	<1.8	<0.3			0.07	

**Table 4:** Recipes.

Research Stage	Aggregate Content [g]	Cement Content [g]	Contents of the Additive [g]	Water Content [g]
Control mix	792	352	0	165.44
Stage 1 10% additives	792	316.8	35.2	165.44
Stage 2 20% additions	792	281.6	70.4	165.44

**Table 5:** Symbols of research samples.

Mineral Supplement	Additive Content 10%	Additive Content 20%
No add-on	CEM100	
Compacted microsilica	MkZ10	MkZ20
White microsilica	MkB10	MkB20
Limestone flour	MW10	MW20
Glass flour	MS10	MS20
Basalt dust	PB10	PB20
Glass granulates	GS10	GS20

**Table 6:** Reactivity categories.

Aggregate Reactivity Category	Descriptive Term	14-day Sample Length Change [%]
R0	Non-reactive	≤0.15
R1	Moderately reactive	>0.15; ≤0.30
R2	Highly reactive	>0.30; ≤0.45
R3	Very strongly reactive	>0.45

**Table 7:** Percentage change in length of samples for Stage 1.

Sample	L1	L4	L7	L10	L14	L16
CEM100 P1	0.0139	0.1010	0.1847	0.2866	0.3290	0.3861
CEM100 P2	0.0154	0.0998	0.1733	0.2820	0.3340	0.3879
CEM100 P3	0.0143	0.0102	0.1798	0.2877	0.3301	0.3841
PB10 P1	0.0158	0.0939	0.1497	0.2162	0.2520	0.2939
PB10 P2	0.0158	0.0949	0.1475	0.2067	0.2513	0.2905
PB10 P3	0.0152	0.0944	0.1395	0.2094	0.2531	0.2919
MS10 P1	0.0046	0.0773	0.1211	0.1765	0.2134	0.2519
MS10 P2	0.0139	0.0789	0.1131	0.1801	0.2174	0.2544
MS10 P3	0.0099	0.0781	0.1205	0.1792	0.2184	0.2551
GS10 P1	0.0081	0.0737	0.1627	0.2099	0.2495	0.2959
GS10 P2	0.0138	0.0796	0.1419	0.2100	0.2492	0.2996
GS10 P3	0.0124	0.0762	0.1554	0.2105	0.2509	0.2967
MkB10 P1	0.0038	0.0162	0.0139	0.0289	0.0500	0.0851
MkB10 P2	0.0046	0.0158	0.0204	0.0324	0.0574	0.0944
MkB10 P3	0.0040	0.0166	0.0184	0.0304	0.0541	0.0911
MW10 P1	0.0197	0.0779	0.1384	0.2112	0.2386	0.2837
MW10 P2	0.0196	0.0834	0.1415	0.2126	0.2422	0.2888
MW10 P3	0.0184	0.0845	0.1378	0.2100	0.2377	0.2856
MkZ10 P1	0.0539	0.2674	0.3035	0.3551	0.3778	0.4216
MkZ10 P2	0.0639	0.2854	0.3100	0.3612	0.3809	0.4240
MkZ10 P3	0.0601	0.2771	0.3065	0.3593	0.3812	0.4221

**Table 8:** Percentage change in length of samples for Stage 2.

Sample	L1	L4	L7	L10	L14	L16
CEM100 P1	0.0139	0.1010	0.1847	0.2866	0.3290	0.3861
CEM100 P2	0.0154	0.0998	0.1733	0.2820	0.3340	0.3879
CEM100 P3	0.0143	0.0102	0.1798	0.2877	0.3301	0.3841
PB20 P1	0.0088	0.0719	0.1100	0.1496	0.1888	0.2122
PB20 P2	0.0058	0.0642	0.1011	0.1457	0.1822	0.2064
PB20 P3	0.0074	0.0699	0.1085	0.1421	0.1723	0.2044
MS20 P1	0.0092	0.0488	0.0757	0.1096	0.1299	0.1703
MS20 P2	0.0100	0.0458	0.0673	0.0989	0.1208	0.1546
MS20 P3	0.0110	0.0399	0.0686	0.1160	0.1235	0.1599
GS20 P1	0.0119	0.0651	0.0924	0.1414	0.1645	0.2011
GS20 P2	0.0062	0.0620	0.0905	0.1432	0.1644	0.2002
GS20 P3	0.0091	0.0701	0.0899	0.1451	0.1654	0.2023
MkB20 P1	0.0004	0.0027	-0.0058	0.0058	0.0069	0.0166
MkB20 P2	0.0008	0.0015	-0.0042	0.0027	0.0046	0.0154
MkB20 P3	0.0005	0.0021	-0.0056	0.0047	0.0051	0.0171
MW20 P1	0.0100	0.0758	0.1340	0.1848	0.2136	0.2514
MW20 P2	0.0108	0.0819	0.1416	0.1946	0.2254	0.2573
MW20 P3	0.0116	0.0901	0.1399	0.1908	0.2213	0.2605
MkZ20 P1	0.0169	0.1047	0.2341	0.2868	0.3465	0.4224
MkZ20 P2	0.0196	0.0943	0.3290	0.3979	0.4518	0.5149
MkZ20 P3	0.0184	0.1005	0.2667	0.3658	0.4298	0.4654

## Data Availability

The original contributions presented in this study are included in the article. Further inquiries can be directed to the corresponding author.
